# Association Between Changes in Physical Activity and New-Onset Atrial Fibrillation After ICD/CRT-D Implantation

**DOI:** 10.3389/fcvm.2021.693458

**Published:** 2021-08-26

**Authors:** Xuerong Sun, Shuang Zhao, Keping Chen, Wei Hua, Yangang Su, Wei Xu, Fang Wang, Xiaohan Fan, Yan Dai, Zhimin Liu, Shu Zhang

**Affiliations:** ^1^Arrhythmia Center, State Key Laboratory of Cardiovascular Disease, Fuwai Hospital, National Center for Cardiovascular Diseases, Chinese Academy of Medical Sciences and Peking Union Medical College, Beijing, China; ^2^Department of Cardiology, Shanghai Institute of Cardiovascular Diseases, Zhongshan Hospital, Fudan University, Shanghai, China; ^3^Department of Cardiology, Nanjing Drum Tower Hospital, Nanjing, China; ^4^Department of Cardiology, Shanghai First People's Hospital, Shanghai Jiao Tong University School of Medicine, Shanghai, China

**Keywords:** atrial fibrillation, changes in physical activity, cardiac death, all-cause mortality, implantable cadioverter defibrillators

## Abstract

**Background:** Changes in physical activity (PA) after implantable cardioverter defibrillator (ICD) or cardiac resynchronization therapy defibrillators (CRT-D) implantation were unknown. The association of PA changes with new-onset atrial fibrillation (AF), cardiac death and all-cause mortality was unclear in patients at high risk of sudden cardiac death.

**Methods:** Patients receiving ICD/CRT-D implantation from SUMMIT registry were retrospectively analyzed. Changes in PA were considered from baseline status to 1 year after implantation. New-onset AF was defined as the first atrial high-rate episode ≥1% of the daily AF burden detected after implantation.

**Results:** Over a mean follow-up of 50.3 months, 124 new-onset AF events (36.2%), 61 cardiac deaths (17.8%), and 87 all-cause deaths (25.4%) were observed in 343 patients with ICD/CRT-D implantation. PA at 1 year after implantation was increased compared with PA at baseline (11.97 ± 5.83% vs. 10.82 ± 5.43%, *P* = 0.008), and PA at 1 year was improved in 210 patients (61.2%). Per 1% decrease in PA was associated with 12.4, 18.3, and 14.3% higher risks of new-onset AF, cardiac death and all-cause mortality, regardless of different baseline characteristics. Patients with decreased PA had 2-fold risks of new-onset AF (hazard ratio [HR] = 1.972, 95% confidence interval [CI]: 1.352–2.877, *P* < 0.001) as high as those with unchanged/increased PA. Decreased PA was an independent risk factor for cardiac death (HR = 3.358, 95% CI: 1.880–5.996, *P* < 0.001) and all-cause mortality (HR = 2.803, 95% CI:1.732–4.535, *P* < 0.001).

**Conclusion:** PA decrease after ICD/CRT-D implantation is associated with a higher incidence of new-onset AF, resulting in worsened outcomes in cardiac death and all-cause mortality.

## Introduction

Atrial fibrillation (AF) is a common cardiac arrhythmia with an increasing prevalence (1). It was reported the prevenance of AF in patients with implantable cardioverter defibrillators (ICDs) was as high as 25% (2). AF is associated with higher risks of ischemic stroke, cardiovascular events, hospitalizations, and all-cause mortality (3, 4). In patients receiving ICD or cardiac resynchronization therapy defibrillator (CRT-D) implantation, new-onset AF was also associated with a greater number of ICD shocks for ventricular arrhythmia, inappropriate shocks, hospitalizations for heart failure (HF), and increased mortality (5–8). Thus, it is essential to predict the incidence of new-onset AF and initiate anticoagulation and rate control management to improve the long-term clinical outcomes after ICD/CRT-D implantation.

Physical activity (PA) can predict the outcomes of different diseases (9–12). PA can be measured via questionnaires to reflect an individual's functional status over the preceding years or months (13). Moreover, accelerometer-derived PA can be detected within the first 30–60 days to reflect the baseline PA status (14). Low levels of baseline PA were associated with higher incidences of hospitalizations for HF, cardiac death, and all-cause mortality after ICD/CRT-D implantation (11, 14). Baseline PA can also predict new-onset AF among the general population or patients with HF (15–18). Chelu et al. found PA decreased and mortality increased significantly after a newly persistent AF episode in patients with ICD (19). It was indicated that longitudinal decrease in PA was associated with a higher incidence of new-onset AF, resulting in worsened long-term outcomes.

To date, few studies have assessed changes in PA after ICD/CRT-D implantation. In the present study, early changes in PA from baseline to 1 year after implantation were evaluated in patients with ICD/CRT-D implantation. Dual-chamber ICDs can provide quantitative and continuous daily PA and AF burden data using a remote home monitoring system, enabling accurate detection of PA changes and new-onset AF (15). This study aimed to investigate the association of changes in PA with the incidence of new-onset AF, as well as explore the effects of PA decreases on long-term cardiac death and all-cause mortality among patients at high risk of sudden cardiac death.

## Methods

The Biotronik Home Monitoring System Safety and Efficacy in Cardiac Implantable Electronic Device-implanted Patients (SUMMIT) registry is a prospective, observational, and multicentre study in China. We performed a retrospective analysis using the archived home monitoring transmission data from SUMMIT registry and evaluated the association of changes in PA with the incidence of new-onset AF, long-term cardiac death, and all-cause mortality. The present study, which conformed to the Declaration of Helsinki, was approved by all participating institutions. All patients provided written informed consent before entering this study.

### Study Participants

Patients who underwent ICD/CRT-D implantation between May 2010 and May 2015 were included when the following criteria were met: (1) ICD or CRT-D was implanted in accordance with the current guideline's recommendations; (2) continuous home monitoring transmission started immediately after implantation; (3) data related to AF daily burden and PA were available; (4) patients were aged ≥18 years at implantation; and (5) life expectancy was >1 year after device implantation. Patients were excluded when (1) AF, atrial flutter (AFL) or atrial tachycardia (AT) was diagnosed using ICD-10 codes (ICD-10: I48, I49.9) before enrolment; (2) patients were lost to follow-up; (3) remote monitoring transmission data was missing or incomplete; or (4) patients were diagnosed with a malignant tumor or scheduled for heart transplantation.

### Measurement of AF Daily Burden and PA

The Biotronik remote home monitoring system can transmit and store data from implantable devices to service centers every day. Data on daily AF burden and PA were collected for each patient. Daily AF burden was expressed as a percentage of the time with atrial high-rate episodes (AHREs) > 180 beats in a 24-h period, where 1% indicated a total of 14.4 min of AHREs per day, which is the minimum daily AF burden. Similar measurement algorithms were previously shown to have a sensitivity and specificity of 95% for AHREs and AF burden detection (20, 21).

Continuous PA was recorded using a Biotronik accelerometer sensor. PA was defined as a percentage of total daily duration recorded when rates were higher than basic rates, where 10% indicated 2.4 h of daily PA, with a resolution of 2 s. PA at baseline was the average daily value detected during the first 30–60 days after ICD/CRT-D implantation, as recommended by previous studies (14), and PA at 1 year was detected during the first 30 days at 1 year after ICD/CRT-D implantation.

### Study Endpoints and Follow-Up

The primary endpoint was new-onset AF. New-onset AF was defined as the first AHRE ≥1% of the daily AF burden, whether symptomatic or not, detected after ICD/CRT-D implantation (15, 20).

The secondary endpoints were cardiac death (ICD-10: I00 to I09, I11, and I20 to I51) and all-cause mortality. The date and cause of death was based on the death certificate. Routine follow-ups were conducted via telephone or clinics. If data transmission was disrupted, the clinical research coordinator immediately confirmed the patient's conditions by contacting the family members.

### Data Collection

Baseline data for the enrolled patients were derived from medical records during hospitalization, including age at implantation, sex, body mass index (BMI), ICD or CRT-D implantation, New York Heart Association (NYHA) class, echocardiographic characteristics (left ventricular ejection fraction [LVEF] and left ventricular end diastolic diameter [LVEDD]), comorbidities [hypertension, diabetes mellitus [DM], stroke, dilated cardiomyopathy [DCM], hypertrophic cardiomyopathy [HCM], ischemic cardiomyopathy [ICM], valvular disease, prior myocardial infarction [MI], percutaneous coronary intervention [PCI], and pre-implant syncope], and medication (angiotensin-converting enzyme inhibitors or angiotensin receptor blockers [ACEIs/ARBs], diuretics, aldosterone antagonists, calcium channel blockers [CCBs], statins, beta-blockers, amiodarone and antiplatelets).

### Grouping

Changes in PA were considered from baseline to 1 year after ICD/CRT-D implantation. All enrolled patients were divided into three groups based on the tertiles of changes in PA, including PA decreased group (Tertile 1, *n* = 114, mean = −2.85%, range [−12.9 to −0.4%]), PA unchanged group (Tertile 2, *n* = 114, mean = 0.98%, range [−0.4–2.2%]), and PA increased group (Tertile 3, *n* = 115, mean = 5.29%, range [2.3–18.0%]).

### Statistical Methods

Continuous variables are presented as means ± standard deviations (SDs), and categorical variables are presented as frequencies and percentages. Clinical characteristics were compared across decreased PA, unchanged PA, and increased PA groups using one-way analysis of variance for continuous variables and a chi-square test for categorical variables. Clinical outcomes, including new-onset AF, long-term cardiac death, and all-cause mortality, were calculated and compared using chi-square test.

Considering the competing risks, cumulative incidence function and Fine and Gray model were used in the survival analysis of detected new-onset AF between different groups of PA performance. Kaplan-Meier survival curves and cox proportional hazards regression models were performed to evaluate the association of changes in PA with cardiac death and all-cause mortality. In the multivariate analysis, model 1 was adjusted for age at implantation and sex. Model 2 was adjusted for additional confounders, including BMI, LVEF, LVEDD, ICD or CRT-D implantation, NYHA class, hypertension, DM, DCM, ICM, prior MI, PCI, and ACEI/ARB, diuretics, and aldosterone antagonist usage. Model 3 was adjusted for the above-mentioned confounders and baseline PA.

Subgroup analysis was performed to explore the effects of changes in PA on the incidence of new-onset AF in patients with different baseline characteristics using Fine & Gray model. Hazard ratios (HRs) and 95% confidence intervals (CIs) were calculated to show the impact. *P*-values < 0.05 were considered statistically significant. Statistical analyses were conducted using SPSS Statistics version 23.0 (IBM Corp., Armonk, NY) and R version 4.0.3 (Bunny-Wunnies Freak Out, The R Foundation for Statistical Computing, Vienna, Austria).

## Results

### Baseline Characteristics

A total of 1,015 patients with ICD/CRT-D implantation were retrospectively analyzed. Patients were excluded due to lack of data on AF daily burden (*n* = 520), unavailable or incomplete monitoring data (*n* = 75), <1 year survival after device implantation (*n* = 40), and diagnosis of AF and/or AFL before enrolment (*n* = 37, 36 AF cases and 1 AFL case). Finally, a total of 343 patients were included in the study.

Overall, PA at 1 year after ICD/CRT-D implantation was significantly higher than PA at baseline (11.97 ± 5.83 vs. 10.82 ± 5.43%, *P* = 0.008). PA was improved at 1 year in 210 patients (61.2%), compared with baseline PA. Changes in PA across PA decreased, PA unchanged, and PA increased groups were −2.85 ± 2.63, 0.98 ± 0.79, and 5.29 ± 2.93%, respectively. The average age at implantation was 62.53 ± 13.54 years, and male patients were dominant in the cohort study (77.6%). The mean LVEF and LVEDD were 39.08 ± 14.57% and 62.07 ± 13.37 mm, respectively. About half of the patients (51.0%) underwent CRT-D implantation. Significant differences between the groups were observed for baseline PA (*P* < 0.001) and PA at 1 year (*P* < 0.001), in addition to sex (*P* = 0.042), and PCI (*P* = 0.039). No significant differences were observed for other baseline characteristics. Table 1 illustrates the comparison of baseline characteristics across three groups.

**Table 1:** Baseline characteristics.

	**Total**	**PA decreased group**	**PA unchanged group**	**PA increased group**	***P*-value**
	***N* = 343**	**(PA changes tertile 1, *n* = 114)**	**(PA changes tertile 2, *n* = 114)**	**(PA changes tertile 3, *n* = 115)**	
**Physical performance**
PA at baseline, %	10.82 ± 5.43	12.59 ± 5.54	9.98 ± 5.02	9.90 ± 5.33	<0.001
PA at 1 year, %	11.97 ± 5.83	9.74 ± 5.38	10.96 ± 5.08	15.18 ± 5.58	<0.001
Changes in PA, %	1.15 ± 4.06	−2.85 ± 2.63	0.98 ± 0.79	5.29 ± 2.93	–
**Demographic characteristics**
Age at implantation, years	62.53 ± 13.54	63.56 ± 12.94	62.63 ± 14.14	61.41 ± 13.55	0.484
Sex, male%	266 (77.6%)	93 (81.6%)	93 (81.6%)	80 (69.6%)	0.042
BMI, Kg/m^2^	23.62 ± 2.84	23.91 ± 2.80	23.74 ± 2.80	23.20 ± 2.90	0.146
CRT-D implantation	175 (51.0%)	59 (51.8%)	58 (50.9%)	58 (50.4%)	0.980
NYHA class III-IV	209 (60.9%)	69 (60.5%)	71 (62.3%)	69 (60.0%)	0.934
**Echocardiological characteristics**
LVEF, %	39.08 ± 14.57	39.75 ± 13.89	38.63 ± 15.51	38.87 ± 14.38	0.830
LVEDD, mm	62.07 ± 13.37	63.15 ± 13.06	62.71 ± 14.70	60.39 ± 12.21	0.252
**Comorbidity**
Hypertension	121 (35.3%)	48 (42.1%)	40 (35.1%)	33 (28.7%)	0.105
DM	48 (14.0%)	20 (17.5%)	18 (15.8%)	10 (8.7%)	0.124
Stroke	8 (2.3%)	4 (3.5%)	2 (1.8%)	2 (1.7%)	0.595
DCM	100 (29.2%)	33 (28.9%)	33 (28.9%)	34 (29.6%)	0.993
HCM	12 (3.5%)	6 (5.3%)	4 (3.5%)	2 (1.7%)	0.349
ICM	131 (38.2%)	47 (41.2%)	40 (35.1%)	44 (38.3%)	0.634
Valvular disease	9 (2.6%)	4 (3.5%)	5 (4.4%)	0 (0.0%)	0.089
Prior MI	52 (15.2%)	21 (18.4%)	13 (11.4%)	18 (15.7%)	0.330
PCI	38 (11.1%)	19 (16.7%)	7 (6.1%)	12 (10.4%)	0.039
Pre-implant syncope	58 (16.9%)	13 (11.4%)	22 (19.3%)	23 (20.0%)	0.157
**Medication**
ACEIs/ARBs	141 (41.1%)	48 (42.1%)	45 (39.5%)	48 (41.7%)	0.909
Diuretics	110 (32.1%)	38 (33.3%)	32 (28.1%)	40 (34.8%)	0.520
Aldosterone antagonists	150 (43.7%)	44 (38.6%)	51 (44.7%)	55 (47.8%)	0.358
CCBs	38 (11.1%)	12 (0.5%)	11 (9.6%)	15 (13.0%)	0.697
Statins	77 (22.4%)	26 (22.8%)	22 (19.3%)	29 (25.2%)	0.559
Betablockers	201 (58.6%)	66 (57.9%)	70 (61.4%)	65 (56.5%)	0.742
Amiodarone	95 (27.7%)	30 (26.3%)	27 (23.7%)	38 (33.0%)	0.264
Antiplatelets	78 (22.7%)	26 (22.8%)	29 (25.4%)	23 (20.0%)	0.617

*ACEI/ARB, angiotensin-converting enzyme inhibitors or angiotensin receptor blockers; BMI, body mass index; CCBs, calcium channel blockers; CRT-D, cardiac resynchronization therapy defibrillators; DCM, hypertrophic cardiomyopathy; DM, diabetes mellitus; HCM, hypertrophic cardiomyopathy; ICM, ischemic cardiomyopathy; LVEDD, left ventricular end diastolic diameter; LVEF, left ventricular ejection fraction; MI, myocardial infarction; NYHA class, New York Heart Association class; PA, physical activity*.

### Clinical Outcomes

**Figure 1:**
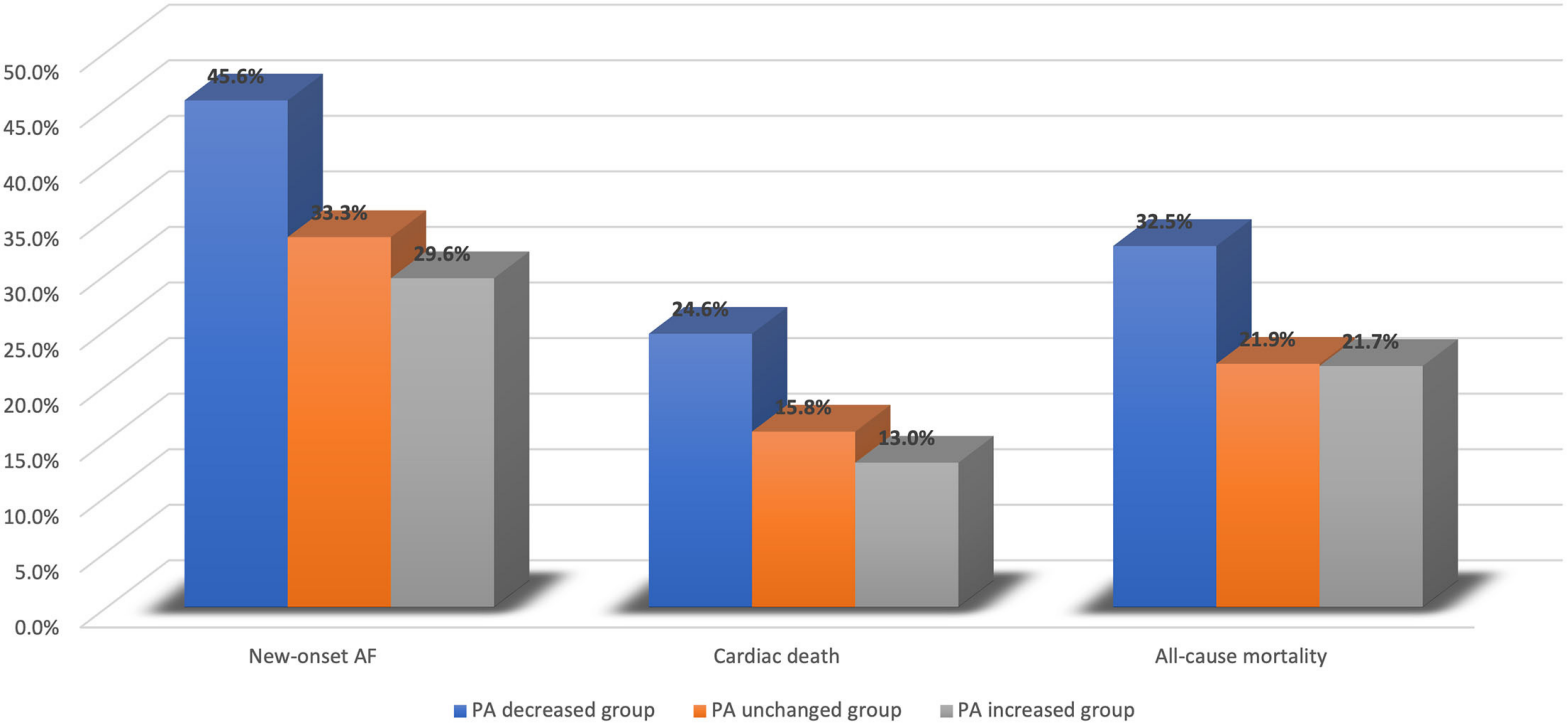
Clinical outcomes across PA decreased group, PA unchanged group, and PA increased group. AF, atrial fibrillation; PA, physical activity.

The average follow-up time was 50.28 ± 17.75 months. A total of 124 new-onset AF events (36.2%) were detected. The incidence of new-onset AF was significantly higher in the PA decreased group than in PA unchanged and PA increased groups (45.6 vs. 33.3 vs. 29.6%, *P* = 0.031). For the secondary endpoints, 61 (17.8%) cardiac deaths and 87 (25.4%) all-cause mortality events occurred. In 124 patients who experienced new-onset AF, 44 death events (35.5%) were observed, which was significantly higher than the incidence of mortality (43/219,19.6%) in the remaining 219 patients who did not experience new-onset AF. Patients in PA decreased group had higher risks of cardiac death (24.6 vs. 15.8 vs. 13.0%, *P* = 0.059) and all-cause mortality (32.5 vs. 21.9 vs. 21.7%, *P* = 0.103) than those in PA unchanged and PA increased groups. Figure 1 illustrates the differences in clinical outcomes across three groups.

### Univariate Survival Analysis for PA Changes and Clinical Outcomes

**Figure 2:**
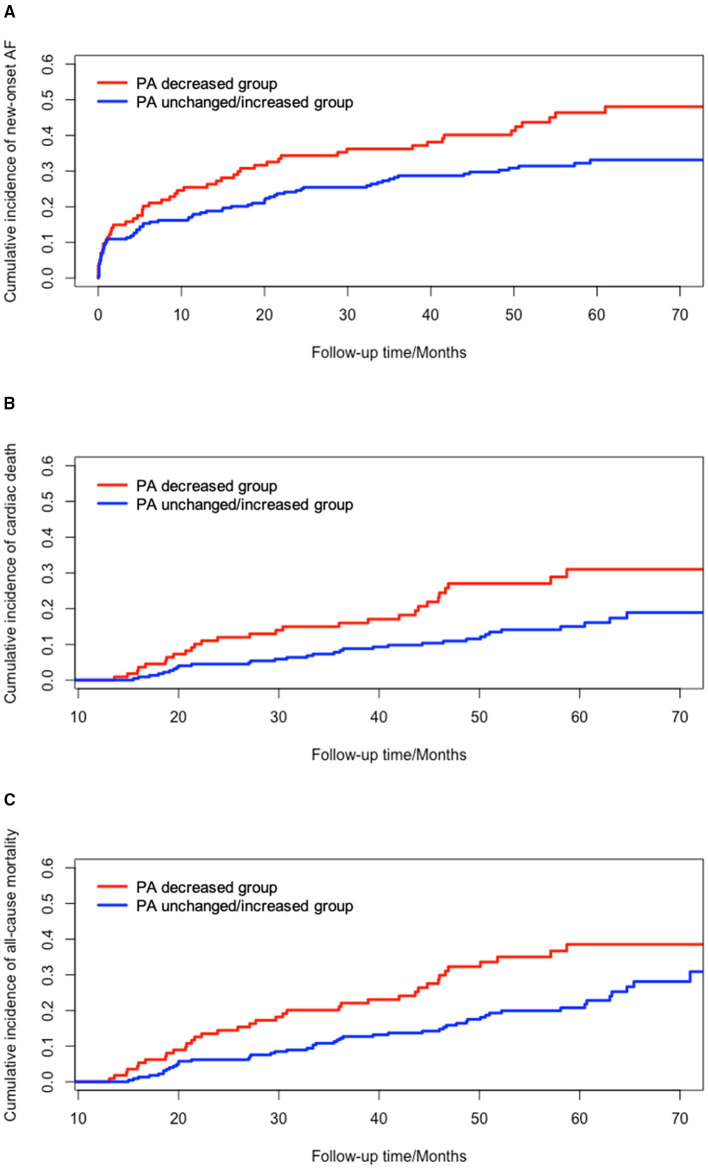
Univariate survival analysis of changes in PA and clinical outcomes. **(A)** cumulative incidence of new-onset AF between PA decreased group and PA unchanged/increased group (*P* = 0.013); **(B)** cumulative incidence of cardiac death between PA decreased group and PA unchanged/increased group (*P* = 0.007); **(C)** cumulative incidence of all-cause mortality between PA decreased group and PA unchanged/increased group (*P* = 0.010). AF, atrial fibrillation; PA, physical activity.

Cumulative incidence function and Kaplan-Meier survival curves were plotted to compare the cumulative incidences of new-onset AF, long-term cardiac death, and all-cause mortality between the PA decreased and unchanged/increased groups. Univariate analysis demonstrated that patients had significantly higher incidences of new-onset AF (*P* = 0.013), cardiac death (*P* = 0.007), and all-cause mortality (*P* = 0.010) in PA decreased group, compared to PA unchanged/increased group (Figure 2).

### Association of Changes in PA With New-Onset AF

Changes in PA were inversely associated with the incidence of new-onset AF in patients who underwent ICD/CRT-D implantation (Table 2). In Fine and Gray model 1, which was adjusted for age at implantation and sex, changes in PA were inversely associated with the incidence of new-onset AF (HR = 1.073, 95%CI: 1.027–1.121, *P* = 0.002), and PA decreased group was associated with a higher incidence of new-onset AF (HR = 1.558, 95% CI: 1.091–2.227 *P* = 0.015) than PA unchanged/increased group. After adjusting for additional confounders, including BMI, LVEF, LVEDD, ICD or CRT-D implantation, NYHA class, hypertension, DM, DCM, ICM, prior MI, PCI, ACEI/ARB, diuretics, and aldosterone antagonist usage in model 2, and the above-mentioned factors and baseline PA in model 3, PA decrease remained an independent risk factor of new-onset AF. Per 1% decrease in PA at 1 year could result in 12.4% higher risks of new-onset AF (HR = 1.124, 95%CI: 1.069–1.182, *P* < 0.001), and patients with decreased PA had 2-fold risks of new-onset AF (HR = 1.972, 95%CI: 1.352–2.877, *P* < 0.001) as high as those with unchanged/increased PA.

**Table 2:** Changes in PA associated with clinical outcomes.

	**Model 1**		**Model 2**		**Model 3**	
	**HR (95% CI)**	***P*-value**	**HR (95% CI)**	***P*-value**	**HR (95% CI)**	***P*-value**
**New-onset AF**
Changes in PA (per 1%/decrease)^*^	1.073 (1.027–1.121)	0.002	1.088 (1.039–1.138)	<0.001	1.124 (1.069–1.182)	<0.001
PA decreased vs. unchanged/increased	1.558 (1.091–2.227)	0.015	1.679 (1.165–2.419)	0.005	1.972 (1.352–2.877)	<0.001
**Cardiac death**
Changes in PA (per 1%/decrease)^*^	1.093 (1.026–1.166)	0.006	1.106 (1.034–1.183)	0.004	1.183 (1.093–1.280)	<0.001
PA decreased vs. unchanged/increased	1.980 (1.191–3.292)	0.008	2.278 (1.332–3.894)	0.003	3.358 (1.880–5.996)	<0.001
**All-cause mortality**
Changes in PA (per 1%/decrease)^*^	1.064 (1.007–1.131)	0.027	1.079 (1.018–1.143)	0.010	1.143 (1.071–1.221)	<0.001
PA decreased vs. unchanged/increased	1.714 (1.117–2.631)	0.014	1.932 (1.236–3.020)	0.004	2.803 (1.732–4.535)	<0.001

*Fine and Gray for new-onset AF or Cox regression model 1 for cardiac death/all-cause mortality was adjusted for age at implantation and sex. Model 2 was adjusted for additional confounders, including BMI, LVEF, LVEDD, ICD or CRT-D implantation, NYHA class, hypertension, DM, DCM, ICM, prior MI, PCI, ACEI/ARB, diuretics, and aldosterone antagonist usage. Model 3 was adjusted for the above mentioned variables and baseline PA. ACEI, angiotensin-converting enzyme inhibitor; AF, atrial fibrillation; ARB, angiotensin receptor blocker; BMI, body mass index; CI, confidence interval; CRT-D, cardiac resynchronization therapy defibrillator; DM, diabetes mellitus; HR, hazard ratio; ICD, implantable cardioverter defibrillator; ICM, ischemic cardiomyopathy; LVEDD, left ventricular end diastolic diameter; LVEF, left ventricular ejection fraction; MI, myocardial infarction; NYHA class, New York Heart Association class; PA, physical activity; PCI, percutaneous coronary intervention.*

* *Changes in PA was analyzed as a continuous variable and expressed as per 1% decrease in PA changes*.

### Association of Changes in PA With Cardiac Death and All-Cause Mortality

Regarding the long-term cardiac death and all-cause mortality, PA decrease was shown as independent risk predictors (Table 2). Decreased PA was associated with higher risks of cardiac death (HR = 1.980, 95% CI: 1.191–3.292, *P* = 0.008) and all-cause mortality (HR = 1.714, 95% CI: 1.117–2.631, *P* = 0.014), compared to unchanged/increased PA, in multivariate Cox regression model 1, adjusted for age at implantation and sex. After adjusted for age at implantation, sex, BMI, LVEF, LVEDD, ICD or CRT-D implantation, NYHA class, hypertension, DM, DCM, ICM, prior MI, PCI, prior AF and ACEI/ARB, diuretics, and aldosterone antagonist usage, changes in PA remained an independent predictor for cardiac death and all-cause mortality in model 2. After further adjusting for baseline PA in model 3, the results remained consistent for cardiac death and all-cause mortality. Per 1% decrease in PA contributed to 18.3% and 14.3% higher risks in cardiac death (HR = 1.183, 95%CI: 1.093–1.280, *P* < 0.001) and all-cause mortality (HR = 1.143, 95%CI: 1.071–1.221, *P* < 0.001), respectively. Compared to patients with unchanged/increased PA, the risks of cardiac death (HR = 3.358, 95%CI: 1.880–5.996, *P* < 0.001) and all-cause mortality (HR = 2.803, 95%CI: 1.732–4.535, *P* < 0.001) increased 2.4 times and 1.8 times, respectively, in patients with decreased PA.

### Subgroup Analysis Based on Different Baseline Characteristics

Subgroup analysis was performed using Fine and Gray model 3 to evaluate the association of new-onset AF with changes in PA based on different baseline characteristics, including baseline PA (low level <10.2% or high level ≥10.2%), age at implantation (<60 years old or ≥60 years old), BMI (<24 kg/m^2^ or ≥24 kg/m^2^), device type (ICD or CRT-D), and LVEF (<35% or ≥35%) (Table 3). Changes in PA, as a continuous variable, were inversely associated with the incidence of new-onset AF among groups with different baseline characteristics, except for patients with low levels of baseline PA (HR = 1.053, 95%CI: 0.973–1.139, *P* = 0.200).

**Table 3:** Subgroup analysis of new-onset AF based on different characteristics.

Changes in PA^*^	**No. of incident events (n)**	**No. of group participants(N)**	**Model 3**	
**(Per 1%/decrease)**			**HR (95% CI)**	***P*-value**
Low baseline PA (<10.2%)	69 (40.4%)	171	1.053 (0.973–1.139)	0.200
High baseline PA (≥10.2%)	55 (32.0%)	172	1.133 (1.064–1.207)	<0.001
Age at implantation <60 years old	54 (38.0%)	142	1.074 (1.005–1.149)	0.036
Age at implantation ≥60 years old	70 (34.8%)	201	1.153 (1.068–1.245)	<0.001
BMI <24 Kg/m^2^	70 (36.5%)	192	1.111 (1.033–1.195)	0.005
BMI ≥ 24 Kg/m^2^	54 (35.8%)	151	1.124 (1.048–1.206)	0.001
CRT-D implantation	67 (38.3%)	175	1.094 (1.028–1.165)	0.005
ICD implantation	57 (33.9%)	168	1.165 (1.067–1.273)	0.001
LVEF <35%	63 (37.1%)	170	1.133 (1.061-1.210)	<0.001
LVEF ≥ 35%	61 (35.3%)	173	1.104 (1.016–1.201)	0.020

*Fine and Gray Model 3 was adjusted for age at implantation, sex, BMI, LVEF, LVEDD, ICD or CRT-D implantation, NYHA class, hypertension, DM, DCM, ICM, prior MI, PCI, ACEI/ARB, diuretics, and aldosterone antagonist usage and baseline PA. ACEI, angiotensin-converting enzyme inhibitor; AF, atrial fibrillation; ARB, angiotensin receptor blocker; BMI, body mass index; CI, confidence interval; CRT-D, cardiac resynchronization therapy defibrillator; DM, diabetes mellitus; HR, hazard ratio; ICD, implantable cardioverter defibrillator; ICM, ischemic cardiomyopathy; LVEDD, left ventricular end diastolic diameter; LVEF, left ventricular ejection fraction; MI, myocardial infarction; NYHA class, New York Heart Association class; PA, physical activity; PCI, percutaneous coronary intervention.*

* *Changes in PA was analyzed as a continuous variable and expressed as per 1% decrease in PA changes*.

### Effects of Changes in PA at Different Baseline PA Levels

**Figure 3:**
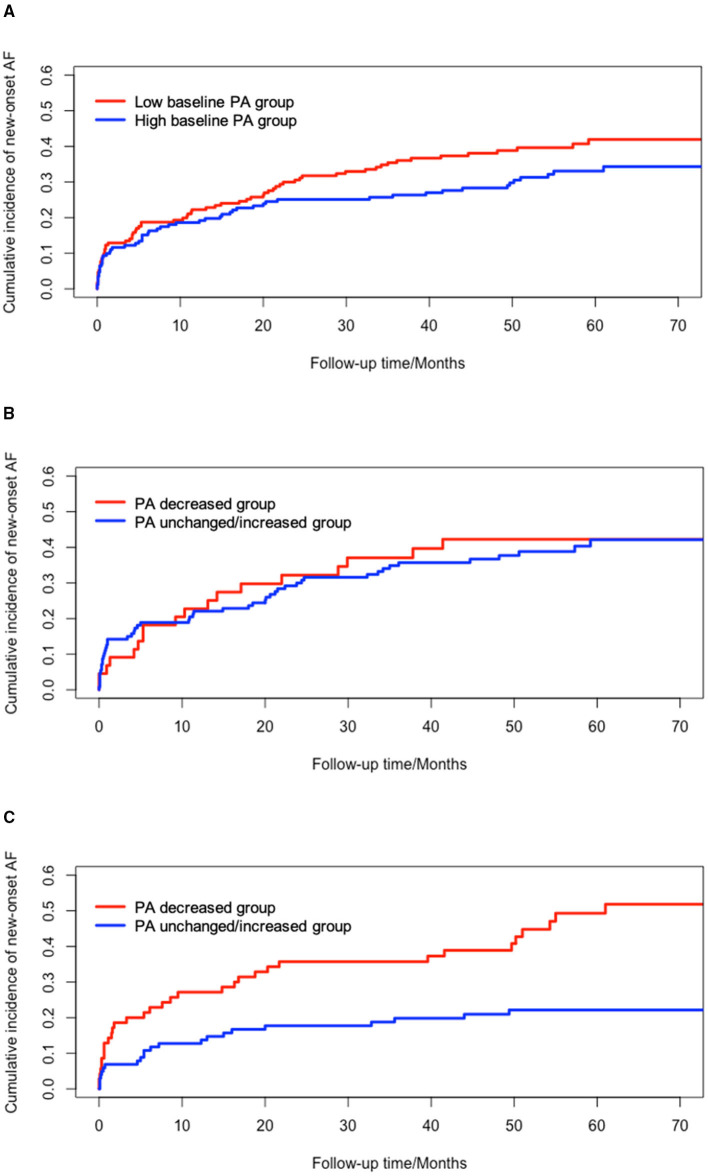
Univariate survival analysis of new-onset AF based on different levels of PA at baseline. **(A)** cumulative incidence of new-onset AF between low baseline PA and high baseline PA group (*P* = 0.117); **(B)** cumulative incidence of new-onset AF in low baseline PA group (*P* = 0.761); **(C)** cumulative incidence of new-onset AF in high baseline PA group (Log-rank, *P* < 0.001). AF, atrial fibrillation; PA, physical activity.

Considering the potential effects of PA at baseline on new-onset AF, the effects of changes in PA was evaluated among patients with high PA at baseline (PA at baseline >10.2%) and low PA at baseline (PA at baseline <10.2%) using cumulative incidence function (Figure 3). Patients with low levels of baseline PA had higher incidence of new-onset AF than those with high levels of baseline PA (*P* = 0.117) although it was not statistically significant. In patients with high baseline PA levels, decreased PA resulted in significant higher incidence of new-onset AF, compared to unchanged/increased PA (*P* < 0.001). However, no significant difference was shown between the PA decreased group and PA unchanged/increased group among patients with low baseline PA levels (*P* = 0.761).

## Discussion

In a cohort study of 343 patients, time-varying changes in PA and new-onset AF were detected using continuous remote monitoring system. Overall, PA at 1 year after ICD/CRT-D implantation was significantly higher than PA at baseline, and PA at 1 year was improved in 210 patients (61.2%). Early changes in PA from baseline to 1 year after implantation were inversely associated with the incidence of new-onset AF, especially among patients with high levels of baseline PA. Additionally, PA decrease at 1 year after implantation remained an independent risk factor of long-term cardiac death and all-cause mortality at 4.2 years.

Some studies have clarified the association of activity performance with AF episodes, hospitalisations, and death among different populations (13, 15–18). The ARIC and MESA studies and a prospective case-control study conducted by Calvo et al. discovered that vigorous PA was associated with a lower risk of incident AF in the general population (16–18). The EORP-AF pilot survey demonstrated that low PA levels were associated with higher risks of cardiovascular death and all-cause mortality in patients with AF, indicating that efforts to increase PA might improve outcomes among AF patients (13). However, PA was measured by patients' self-reported questionnaires, which only indicated the activity level over the preceding years or months, and incident AF episodes were detected using intermittent electrocardiograms. In the IMPLANTED registry (15), objective accelerometer-derived PA and continuous monitoring of AHREs were obtained using remote monitoring system in HF patients who received ICD implantation. It was observed that low baseline PA levels can predict the incidence of AHREs, death, or HF hospitalisations, although the detected AHREs might not be newly AF episodes. For the underlying mechanism between physical performance and AF episodes, vigorous PA probably contributed to weight loss, which has known cardioprotective effects for AF episodes. The CARDIO-FIT Study, Henry Ford Exercise Testing Project, and HUNT3 study reported that high levels of cardiorespiratory fitness (CRF) measured using exercise stress tests were associated with a significantly reduced number of AF episodes among overweight and obese populations over a long-term follow-up (4–8 years) (22–24). However, those above-mentioned studies only focused on baseline PA performance and the individual time-varying PA changes were not discussed.

Regarding the changes in PA, the longitudinal changes in PA over an entire lifespan were expressed as the total energy expenditure by Westerterp KR et al., which showed that PA gradually increased from an early age to adulthood but decreased in old age (25). Vamos M et al. reported that significant short-term decrease in device-measured PA was associated with a high incidence of hospitalisations for HF in CRT-D recipients (26). Decrease in PA was adopted during a 20-day windows period prior to OptiVol alerts, which was proved to be an independent predictor of the following hospitalization for HF (26). The DISCERN AF study demonstrated an association between AF burden and activity level changes in patients after AF ablation. PA began to decrease after the AF daily burden exceeded 500 min (34.7%) and dropped after 1,000 min (69.4%) (27). Although the inverse relationship between AF burden and activity level changes was not shown at an individual level, it was suggested that individual time-varying PA may be a valuable prognostic predictor for the incidence of new-onset AF. Different from these two studies, the present study measured time-varying changes in PA after ICD/CRT-D implantation for each patient, using a continuous, accurate, and rapid monitoring system. We observed that PA at 1 year after device implantation was generally higher than baseline PA (11.97 ± 5.83 vs. 10.82 ± 5.43%, *P* = 0.008), and PA increase was observed in 210 patients (61.2%) with an average value of 3.44 ± 3.00%. This finding was consistent with previous published studies, which showed that PA at 3 and 6 months after CRT implantation were generally higher than baseline PA of patients with HF (28). Regarding the mechanism of PA changes after device implantation, the improvement of PA was likely due to the clinical effects of device implantation or lifestyle modification on activity (28, 29). However, worsening PA might reflect that symptomatic or ongoing AF with heavy AHRE burdens exerted limitations on activity tolerance (25).

The present study focused on new-onset AF events during the whole monitoring period, which were detected by ICD/CRT-D devices. A 36.2% incidence of new-onset AF (AF daily burden ≥14 min) over a follow-up period of 4.2 years was reported, which was comparable to the incidence of newly AHREs (30.1%) detected among patients with HF (15). After adjusting for considerable demographic and echocardiographic characteristics, comorbidities, medication, and baseline PA, PA decrease was demonstrated as a strong independent risk factor of new-onset AF. Additionally, changes in PA remained inversely associated with the incidence of new-onset AF among different groups with various baseline characteristics, except for patients with low levels of baseline PA. The possible explanation might be that less patients at low baseline PA levels had decreased PA, especially when the sample size was not large enough. Low baseline PA could also predict the incident new-onset AF events, probably hiding the association between PA changes and new-onset AF. In the clinical complication, the occurrence of new-onset AF should be considered when PA decreases after implantation, especially for those with good performance of PA at baseline. ECG, anticoagulation, and rate control treatment were required to confirm and manage AF, when necessary. Among patients at high risk of sudden cardiac death, initiating AF management might help to decrease the risks of ICD shocks for ventricular arrhythmias and inappropriate shocks (5). Moreover, patients with PA decrease at 1 year after device implantation had 2–3 times higher risk of long-term cardiac death and all-cause mortality than those with PA increases. Time-varying PA after device implantation was valuable to reflect the clinical response and therapeutic efficacy of device implantation (28). Only focusing baseline PA performance was not enough. This study highlighted the importance of monitoring the time-varying PA changes for improving long-term outcomes. Besides this, it is suggested that exercise rehabilitation might improve long-term clinical outcomes in patients receiving ICD/CRT-D implantation, especially for patients with a high level of PA at baseline.

## Limitation

There are several limitations in the present study. First, the sample size was small, which might limit the analysis of PA changes in low baseline group; Second, the study participants were patients with high risks of sudden cardiac death, so further studies are needed to determine if the association of early changes in PA with new-onset AF can be generalized to other patient populations. Third, due to the limitation of the home monitoring system, AHREs with <1% of total AF burden over a 24-h period cannot be detected. However, the measurement algorithms in this analysis were previously shown to have a sensitivity and specificity of 95% for AHREs and AF burden detection (21).

## Conclusion

PA decrease at 1 year was associated with a higher risk of new-onset AF, regardless of baseline characteristics. Moreover, PA decrease was an independent predictor for long-term cardiac death and all-cause mortality in patients at high risk of sudden cardiac death.

## Data Availability Statement

The original contributions presented in the study are included in the article/supplementary materials, further inquiries can be directed to the corresponding author/s.

## Ethics Statement

The studies involving human participants were reviewed and approved by Fuwai Hospital (the chief institute) and all other participating organizations (Zhongshan Hospital Fudan University, Nanjing Drum Tower Hospital, Shanghai First People's Hospital et al.). The patients/participants provided their written informed consent to participate in this study.

## Author Contributions

XS and SZhao performed the conception or design of the work. XS, KC, WH, YS, WX, FW, XF, YD, ZL, and SZhang contributed to the acquisition, analysis, and interpretation of data for the work. XS drafted the manuscript. SZhao critically revised the manuscript. All gave final approval and agreed to be accountable for all aspects of work ensuring integrity and accuracy.

## Conflict of Interest

The authors declare that the research was conducted in the absence of any commercial or financial relationships that could be construed as a potential conflict of interest.

## Publisher's Note

All claims expressed in this article are solely those of the authors and do not necessarily represent those of their affiliated organizations, or those of the publisher, the editors and the reviewers. Any product that may be evaluated in this article, or claim that may be made by its manufacturer, is not guaranteed or endorsed by the publisher.

## Abbreviations

AADs: antiarrhythmic drugs
ACEI/ARB: angiotensin-converting enzyme inhibitors or angiotensin receptor blockers
AF: atrial fibrillation
AHRE: atrial high-rate episode
BMI: body mass index
CCBs: calcium channel blockers
CI: confidence interval
HR: hazard ratio
CRT-D: cardiac resynchronization therapy defibrillators
DCM: hypertrophic cardiomyopathy
DM: diabetes mellitus
HCM: hypertrophic cardiomyopathy
ICD: implantable cardioverter-defibrillator
ICM: ischemic cardiomyopathy
LVEDD: left ventricular end diastolic diameter
LVEF: left ventricular ejection fraction
MI: myocardial infarction
NYHA class: New York Heart Association class
PA: physical activity
PCI: percutaneous coronary intervention.

## References

[1] Go AS Hylek EM Phillips KA Chang Y Henault LE Selby JV. Prevalence of diagnosed atrial fibrillation in adults: national implications for rhythm management and stroke prevention: the AnTicoagulation and Risk Factors in Atrial Fibrillation (ATRIA) Study. JAMA. (2001) 285:2370–5. 10.1001/jama.285.18.2370 11343485

[2] Botto GL Luzi M Ruffa F Russo G Ferrari G. Atrial tachyarrhythmias in primary and secondary prevention ICD recipients: clinical and prognostic data. Pacing Clin Electrophysiol. (2006) 29:S48–53. 10.1111/j.1540-8159.2006.00489.x 17169133

[3] Steinberg BA Hellkamp AS Lokhnygina Y Patel MR Breithardt G Hankey GJ. Higher risk of death and stroke in patients with persistent vs. paroxysmal atrial fibrillation: results from the ROCKET-AF Trial. Eur Heart J. (2015) 36:288–96. 10.1093/eurheartj/ehu359 25209598 PMC4313363

[4] Wang TJ Larson MG Levy D Vasan RS Leip EP Wolf PA. Temporal relations of atrial fibrillation and congestive heart failure and their joint influence on mortality: the Framingham Heart Study. Circulation. (2003) 107:2920–5. 10.1161/01.CIR.0000072767.89944.6E 12771006

[5] Borleffs CJ Ypenburg C van Bommel RJ Delgado V Erven L Schalij MJ. Clinical importance of new-onset atrial fibrillation after cardiac resynchronization therapy. Heart Rhythm. (2009) 6:305–10. 10.1016/j.hrthm.2008.12.017 19251202

[6] Bunch TJ Day JD Olshansky B Stolen KQ Mullin CM; INTRINSIC RV Study Investigators. Newly detected atrial fibrillation in patients with an implantable cardioverter-defibrillator is a strong risk marker of increased mortality. Heart Rhythm. (2009) 6:2–8. 10.1016/j.hrthm.2008.09.025 18996055

[7] Kleemann T Strauss M Kouraki K Werner N Zahn R. Prognostic relevance of new onset arrhythmia and ICD shocks in primary prophylactic ICD patients. Clin Res Cardiol. (2020) 109:89–95. 10.1007/s00392-019-01491-1 31087157

[8] Vergara P Solimene F D'Onofrio A Pisanò EC Zanotto G Pignalberi C. Are atrial high-rate episodes associated with increased risk of ventricular arrhythmias and mortality? JACC Clin Electrophysiol. (2019) 5:1197–208. 10.1016/j.jacep.2019.06.018 31648745

[9] Elliott AD Linz D Mishima R Kadhim K Gallagher C Middeldorp ME. Association between physical activity and risk of incident arrhythmias in 402 406 individuals: evidence from the UK Biobank cohort. Eur Heart J. (2020) 41:1479–86. 10.1093/eurheartj/ehz897 31951255

[10] Biscaglia S Campo G Sorbets E Ford I Fox KM Greenlaw N. Relationship between physical activity and long-term outcomes in patients with stable coronary artery disease. Eur J Prev Cardiol. (2020) 27:426–36. 10.1177/2047487319871217 31558054

[11] Zhao S Chen K Su Y Hua W Chen S Liang Z. Association between patient activity and long-term cardiac death in patients with implantable cardioverter-defibrillators and cardiac resynchronization therapy defibrillators. Eur J Prev Cardiol. (2017) 24:760–7. 10.1177/2047487316688982 28117620

[12] Waschki B Kirsten A Holz O Müller KC Meyer T Watz H. Physical activity is the strongest predictor of all-cause mortality in patients with COPD: a prospective cohort study. Chest. (2011) 140:331–42. 10.1378/chest.10-2521 21273294

[13] Proietti M Boriani G Laroche C Diemberger I Popescu MI Rasmussen LH. Self-reported physical activity and major adverse events in patients with atrial fibrillation: a report from the EURObservational Research Programme Pilot Survey on Atrial Fibrillation (EORP-AF) General Registry. Europace. (2017) 19:535–43. 10.1093/europace/euw150 28431068

[14] Kramer DB Mitchell SL Monteiro J Jones PW Normand SL Hayes DL. Patient activity and survival following implantable cardioverter-defibrillator implantation: the ALTITUDE activity study. J Am Heart Assoc. (2015) 4:e001775. 10.1161/JAHA.115.001775 25979902 PMC4599410

[15] Palmisano P Guerra F Ammendola E Ziacchi M Luigi Pisanò EC Dell'Era G. Physical activity measured by implanted devices predicts atrial arrhythmias and patient outcome: results of IMPLANTED (Italian Multicentre Observational Registry on Patients With Implantable Devices Remotely Monitored). J Am Heart Assoc. (2018) 7:e008146. 10.1161/JAHA.117.008146 29478022 PMC5866336

[16] Bapat A Zhang Y Post WS Guallar E Soliman EZ Heckbert SR. Relation of physical activity and incident atrial fibrillation (from the Multi-Ethnic Study of Atherosclerosis). Am J Cardiol. (2015) 116:883–8. 10.1016/j.amjcard.2015.06.013 26189040 PMC4554984

[17] Huxley RR Misialek JR Agarwal SK Loehr LR Soliman EZ Chen LY. Physical activity, obesity, weight change, and risk of atrial fibrillation: the Atherosclerosis Risk in Communities study. Circ Arrhythm Electrophysiol. (2014) 7:620–5. 10.1161/CIRCEP.113.001244 24907285 PMC4230341

[18] Calvo N Ramos P Montserrat S Guasch E Coll-Vinent B Domenech M. Emerging risk factors and the dose-response relationship between physical activity and lone atrial fibrillation: a prospective case-control study. Europace. (2016) 18:57–63. 10.1093/europace/euv216 26333377 PMC4739323

[19] Chelu MG Gunderson BD Koehler J Ziegler PD Sears SF. Patient activity decreases and mortality increases after the onset of persistent atrial fibrillation in patients with implantable cardioverter-defibrillators. JACC Clin Electrophysiol. (2016) 2:518–23. 10.1016/j.jacep.2016.01.015 29759876

[20] Shanmugam N Boerdlein A Proff J Ong P Valencia O Maier SK. Detection of atrial high-rate events by continuous home monitoring: clinical significance in the heart failure-cardiac resynchronization therapy population. Europace. (2012) 14:230–7. 10.1093/europace/eur293 21933802 PMC3262405

[21] Purerfellner H Gillis AM Holbrook R Hettrick DA. Accuracy of atrial tachyarrhythmia detection in implantable devices with arrhythmia therapies. Pacing Clin Electrophysiol. (2004) 27:983–92. 10.1111/j.1540-8159.2004.00569.x 15271020

[22] Pathak RK Elliott A Middeldorp ME Meredith M Mehta AB Mahajan R. Impact of CARDIOrespiratory FITness on arrhythmia recurrence in obese individuals with atrial fibrillation: the CARDIO-FIT study. J Am Coll Cardiol. (2015) 66:985–96. 10.1016/j.jacc.2015.06.488 26113406

[23] Qureshi WT Alirhayim Z Blaha MJ Juraschek SP Keteyian SJ Brawner CA. Cardiorespiratory fitness and risk of incident atrial fibrillation: results from the Henry Ford Exercise Testing (FIT) project. Circulation. (2015) 131:1827–34. 10.1161/CIRCULATIONAHA.114.014833 25904645

[24] Garnvik LE Malmo V Janszky I Wisløff U Loennechen JP Nes BM. Physical activity modifies the risk of atrial fibrillation in obese individuals: the HUNT3 study. Eur J Prev Cardiol. (2018) 25:1646–52. 10.1177/2047487318784365 29939081

[25] Westerterp KR. Changes in physical activity over the lifespan: impact on body composition and sarcopenic obesity. Obes Rev. (2018) 19(Suppl. 1):8–13. 10.1111/obr.12781 30511504

[26] Vamos M Nyolczas N Bari Z Bogyi P Muk B Szabo B. Refined heart failure detection algorithm for improved clinical reliability of OptiVol alerts in CRT-D recipients. Cardiol J. (2018) 25:236–44. 10.5603/CJ.a2017.0077 28653309

[27] Proietti R Birnie D Ziegler PD Wells GA Verma A. Postablation atrial fibrillation burden and patient activity level: insights from the DISCERN AF study. J Am Heart Assoc. (2018) 7:e010256. 10.1161/JAHA.118.010256 30486704 PMC6405544

[28] Vegh EM Kandala J Orencole M Upadhyay GA Sharma A Miller A. Device-measured physical activity versus six-minute walk test as a predictor of reverse remodeling and outcome after cardiac resynchronization therapy for heart failure. Am J Cardio. (2014) 113:1523–8. 10.1016/j.amjcard.2014.01.430 24641966

[29] Gold MR Daubert JC Abraham WT Hassager C Dinerman JL Hudnall JH. Implantable defibrillators improve survival in patients with mildly symptomatic heart failure receiving cardiac resynchronization therapy: analysis of the long-term follow-up of remodeling in systolic left ventricular dysfunction (REVERSE). Circ Arrhythm Electrophysiol. (2013) 6:1163–8. 10.1161/CIRCEP.113.000570 24125796

